# Specific loss of GIPR signaling in GABAergic neurons enhances GLP-1R agonist-induced body weight loss

**DOI:** 10.1016/j.molmet.2024.102074

**Published:** 2024-11-26

**Authors:** Jordan Wean, Allison Ho Kowalsky, Rhianna Laker, Sarah Will, Daniel J. Drucker, Christopher J. Rhodes, Randy J. Seeley

**Affiliations:** 1 Department of Surgery, University of Michigan, Ann Arbor, MI, USA; 2 Research and Early Development, Cardiovascular, Renal and Metabolic Diseases, BioPharmaceuticals R&D, AstraZeneca, Gaithersburg, MD, USA; 3 Lunenfeld Tanenbaum Research Institute, Mount Sinai Hospital, Department of Medicine, University of Toronto, Toronto, Canada

**Keywords:** Incretin, GLP-1, GIP, Obesity, Anti-obesity medication, Dual-incretin agonism, Tirzepatide

## Abstract

**Objectives:**

Dual incretin agonists are among the most effective pharmaceutical treatments for obesity and type 2 diabetes to date. Such therapeutics can target two receptors, such as the glucagon-like peptide-1 (GLP-1) receptor and the glucose-dependent insulinotropic polypeptide (GIP) receptor in the case of tirzepatide, to improve glycemia and reduce body weight. Regarding body weight effects, GIPR signaling is thought to involve at least two relevant mechanisms: the enhancement of food intake reduction and the attenuation of aversive effects caused by GLP-1R agonists. Although it is known that dual GLP-1R-GIPR agonism produces greater weight loss than GLP-1R agonism alone, the precise mechanism is unknown.

**Methods:**

To address this question, we used mice lacking GIPR in the whole body, GABAergic neurons, or glutamatergic neurons. These mice were given various combinations of GLP-1R and GIPR agonist drugs with subsequent food intake and conditioned taste aversion measurements.

**Results:**

A GIPR knockout in either the whole body or selectively in inhibitory GABAergic neurons protects against diet-induced obesity, whereas a knockout in excitatory glutamatergic neurons had a negligible effect. Furthermore, we found that GIPR in GABAergic neurons is essential for the enhanced weight loss efficacy of dual incretin agonism, yet, surprisingly, its removal enhances the effect of GLP-1R agonism alone. Finally, GIPR knockout in GABAergic neurons prevents the anti-aversive effects of GIPR agonism.

**Conclusions:**

Our findings are consistent with GIPR research at large in that both enhancement and removal of GIPR signaling are metabolically beneficial. Notably, however, our findings suggest that future obesity therapies designed to modulate GIPR signaling, whether by agonism or antagonism, would be best targeted towards GABAergic neurons.

## Highlights

• Whole-body or GABAergic neuron loss of GIPR signaling enhances GLP-1R agonist-induced weight loss.
• Dual GLP-1R/GIPR agonism requires GIPR signaling in GABAergic neurons for its weight loss superiority over GLP-1R agonism.
• GABAergic GIPR-expressing neurons mediate the response to high-fat diet.
• GABAergic GIPR-expressing neurons mediate GIPR agonist reduction of aversion caused by GLP-1R agonism.

## Graphical abstract

**Figure:**
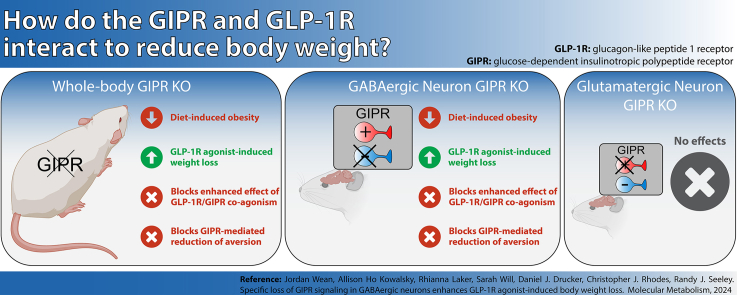

## 1 Introduction

Obesity is increasing in incidence and predicted to affect over 50% of all adults worldwide by 2035 [1]. Being chronically overweight increases the risk of serious disease co-morbidities that, in turn, increase mortality and healthcare costs [2,3]. Behavioral approaches to combat obesity, such as diet and exercise, rarely produce lasting weight loss commonly due to compensatory hyperphagia and hypometabolism [[4], [5], [6], [7], [8]]. Currently, bariatric surgery remains the benchmark for obesity management, but it has risks and is simply not scalable to the number of patients in need of treatment, underscoring the need for more effective pharmacological solutions [9]. Although previous generations of pharmacological weight loss treatments were either ineffective or unsafe, newer incretin-based therapies show significant promise [10,11].

Dual incretin agonists (DIA) have garnered recent attention for their ability to dramatically improve blood glucose levels and promote weight loss. DIAs interact with receptors for both glucagon-like peptide-1 (GLP-1) and glucose-dependent insulinotropic polypeptide (GIP, also known as gastric inhibitory peptide) the incretin hormones with the canonical role of potentiating glucose-stimulated insulin secretion. However, incretin receptors are found throughout the body, and both hormones have numerous extrapancreatic effects [12,13]. Before the creation of DIAs, the most effective non-surgical treatments for obesity were GLP-1 receptor (GLP-1R) monoagonists such as semaglutide, which cause an average weight reduction of about 15 % [14]. In comparison, tirzepatide, currently the only FDA-approved DIA, produced an average weight loss of 21 % or 26 kg [15]. The underlying mechanism for the enhanced efficacy on body weight loss of tirzepatide remains incompletely understood. While the GIP receptor (GIPR) is found in multiple tissues, strong evidence links the added effect of GIPR agonists to actions in the CNS. A whole-brain GIPR KO prevents the enhanced efficacy of DIA treatment on body weight loss, making the CNS the likely target organ [16].

Two hypotheses have been offered to explain the role of CNS GIPRs in the enhanced clinical efficacy of DIAs. The first is that GIPR agonism reduces the aversive effects of a GLP-1R agonist and thereby increases the maximum dose of a GLP-1R agonist that can be clinically tolerated [17]. A variety of evidence demonstrates the ability of GIPR agonists to reduce symptoms of nausea in several species [[18], [19], [20], [21]]. This includes chemogenetic activation of GIPR-expressing neurons that can reduce the aversive effects of both Growth Differentiation Factor-15 (GDF15) receptor agonism and LiCl [20]. However, this explanation has drawbacks. First, in preclinical models where subjects cannot remove themselves from the experiment, such nausea responses likely contribute to initial weight loss. Second, tirzepatide demonstrated significantly increased GI adverse events in the clinical trials studying its efficacy for obesity [15,22]. Hence, it is not clear that at maximally effective doses, a DIA has a superior nausea profile than a GLP-1R agonist alone. The second hypothesis is that agonizing the GIPR reduces food intake which can be additive or synergistic with the effects of a GLP-1R agonist. The body weight loss caused by both GLP-1R and GIPR agonism is mediated through the brain [16,[23], [24], [25]]. However, unlike what is found in pancreatic β-cells, central GIPR and GLP-1R are typically (though not always) found in separate cells [[26], [27], [28], [29]]. This suggests that GLP-1R and GIPR agonism likely work on distinct circuits that either interact directly or project to a common target population. Needless to say, these two hypotheses are not mutually exclusive, and both may contribute to the efficacy of DIAs.

To add to the complexity, treatments that combine GIPR antagonism with GLP-1R agonism can also synergistically enhance the efficacy of GLP-1R agonism to reduce food intake and body weight [30]. This is consistent with genetic data that indicate loss of GIPR signaling results in a lower body mass index (BMI) in humans [31,32], and resistance to weight gain on a high-fat diet (HFD) in mice [[33], [34], [35], [36]]. This resistance to weight gain is recapitulated in mice that lack GIPR signaling exclusively in the CNS [16], and points toward the brain being the crucial target organ for GIPR's role in the regulation of energy balance whether it is via increased or decreased GIPR signaling. One final factor to consider is the possibility that chronic GIPR agonism may result in antagonism. Killion et al. found that chronic GIPR agonism in cultured adipocytes from both mice and humans caused a desensitization to GIP, leading to functional antagonism [37]. While it has not been established that this same phenomenon occurs in neurons, this could explain apparent discrepancies between agonism and antagonism treatments. Notably, mice and humans differ significantly in their response to the GIPR-stimulating actions of tirzepatide, a finding required to properly interpret the data here. Tirzepatide has poor activity at mouse GIPR [38,39]. Thus, we consider the tirzepatide-only drug groups in this study as predominately activating GLP-1R while the tirzepatide + GIPR agonist groups are meant to the mimic full GIPR agonism that a human might experience.

Addressing these crucial mechanistic questions requires the identification of key sets of neurons that express the GIPR and their role in the regulation of energy balance and the response to DIAs. To that end, we developed mice that lack GIPR signaling exclusively in either inhibitory neurons that secrete gamma-aminobutyric acid (GABA) or excitatory neurons that secrete glutamate. For additional comparison, we also generated a whole-body GIPR KO mouse model. Here we report the effect that each model has on drug-induced weight loss and aversive responses.

## 2 Materials and methods

### 2.1 Animals

Rodent experiments were approved by the University of Michigan Institutional Animal Care and Use Committee. Animals were single-housed for a minimum of 1 week prior to experimentation in a temperature controlled room set at the mouse thermoneutrality point (30 °C) on a 12:12 light:dark cycle. Mice were given ad-libitum access to either regular chow (PicoLab 5LOD) or 60% high-fat diet (Research Diets, Inc., D12492). To produce diet-induced obese (DIO) animals, mice were given 60% HFD for at least 8 weeks. Whole body GIPR knockout mice were created by CRISPR-mediated deletion of the GIPR gene. Potential targets for double stranded DNA breaks flanking the DNA region of interest were identified using an online bioinformatic tool Crispor [40]. In GIPR^KO^ mice, confirmation of the knockout was done by measuring GIPR transcripts in white adipose tissue using RT-PCR. No knockout mice had detectable *Gipr* mRNA (Supplementary Figure 1A, n = 4–5). Four guides were tested by zygote injection to confirm DNA cutting. After confirming DNA targeting, the following reagents were injected into 300 C57BL/6 and SJL mixed background zygotes by the University of Michigan Transgenic core: Cas 9 protein (Sigma, 30 ng/μL), Guide RNA (Synthego, 30 ng/μL). Mice were screened for deletion of the gene sequence between the target sites. Founders were crossed to C57BL/6 mice, and offspring were sequenced to confirm inheritance of the mutation. GABAergic and glutamatergic GIPR KO mice were created by crossing either C57BL/6J Vgat-ires-cre (Jackson Laboratories; 028862) or C57BL/6J Vglut-ires-cre (Jackson Laboratories; 028863) knock-in mice with GIPR flox/flox mice [41]. Mice used for preliminary studies in Supplemental Figure 1 were C57BL/6J.

### 2.2 Pharmacological agents

Lyophilized D-Ala^2^-GIP (Tocris, 6699), semaglutide (Bachem, Switzerland), and tirzepatide (Peptide International, Louisville, KY, USA) were reconstituted in saline at 1 mg/mL and frozen at −80 °C. On the day of the experiment, each drug was thawed and diluted with saline to the appropriate concentration and administered subcutaneously. D-Ala^2^-GIP was given at 30 nmol/kg, a dose similar to that used in [42]. Neither 30 nmol/kg nor 150 nmol/kg were sufficient to decrease food intake over 48 h (Supplemental Figure 1B–C, n = 5–6). Additionally, 30 nmol/kg did not cause a conditioned taste aversion (Supplemental Figure 1D, n = 5). Tirzepatide and semaglutide doses (2 nmol/kg) were chosen based off preliminary data so they would be strong enough to decrease food intake and body weight but not so strong to overwhelm any anti-aversive actions of GIPR agonism (Supplemental Figure 1E–G, n = 5–30).

### 2.3 Conditioned taste aversion assay

Mice were handled and injected with saline for a minimum of 3 days before experimentation. During this period, mice were given two water bottles, and access to the lickable water valve was removed to allow them to habituate to drinking from bottles. At onset of the dark cycle on day 4, water bottles were removed for 22 h to induce thirst. On day 5, mice were given both a water bottle and a bottle containing 0.15% saccharin in water for the 2 h before dark cycle onset. Then, mice were injected with either saline (vehicle) or the specified drug, and the saccharin bottle was replaced by water. On day 6 at lights out, water bottles were again removed to induce thirst. On day 7, saccharin and water bottles were returned to the cage, and the bottle weights were measured at 24 h. The preference ratio was calculated as [saccharin intake/(saccharin + water intake)].

### 2.4 Body composition

Body composition (fat and lean mass) was measured using an EchoMRI (Echo Medical Systems).

### 2.5 Chronic food intake and body weight

Mice were dosed daily with the indicated drugs subcutaneously at onset of dark cycle. Food intake and body weight were measured at this time. Mice were excluded from the study if their body weight fell 10% below their pre-dosing lean mass.

### 2.6 Metabolic cages

Mice were placed in metabolic cages (PhenoMaster, TSE Systems) for one week total. These cages measure metabolism via indirect calorimetry, food/water intake, and locomotor/ambulatory activity. The data from the first four days was excluded to allow mice to habituate to their new environment. Data was processed and analyzed with the two group setting in CalR [43]. Meal pattern analysis data was generated by the TSE software with a 0.1 g minimum meal size and 15-min inter-meal interval.

### 2.7 Fast-refeed

For the fast-refeed assay, food was removed from cages 1 h after dark cycle onset for 23 h. At the beginning of dark cycle the next day, food was returned and weighed at hours 0, 1, 2, and 24.

### 2.8 Quantitative real-time PCR

A Qiagen RNeasy isolation kit was used to extract RNA from WAT adipose tissue samples. cDNA was created by reverse transcription from mRNA using a Bio-Rad iScript cDNA synthesis kit. GIPR levels were measured using quantitative real-time RT-PCR using Taqman gene expression assay and was performed using the StepOnePlus detection system (Applied Biosystems) with the standard protocol. Abundance of each transcript was calculated using a standard curve of cycle thresholds and normalized to RL32.

### 2.9 Statistical analysis and figures

Statistical analyses other than ANCOVA were performed in GraphPad Prism v10.0.0 and are represented as means ± SEM. Student's t-test or two-way ANOVA with Bonferroni-corrected post hoc tests were used to determine significance, which was set at p < 0.05. ANCOVA analyses were performed using SPSS 28.0 using the indicated covariates. For paired data in the meal pattern analysis, a paired t-test or paired Wilcoxon signed rank test was used. Figures were created with Prism, Python (Seaborn [44]), and R (ggplot2 [45]).

## 3 Results

### 3.1 Phenotypic characterization

**Figure 1:**
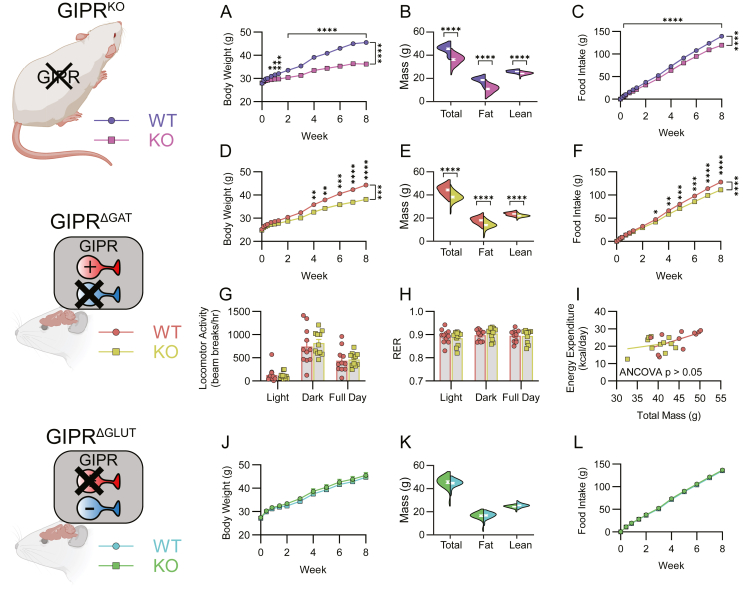
GIPR^KO^ and GIPR^ΔGAT^ are protective against diet-induced obesity. (A) Body weight progression of GIPR^KO^ mice over 8 weeks of HFD feeding with (B) subsequent body composition measurements (dot and white bars indicate mean ± SEM). (C) Food intake measurements for the GIPR^KO^ mice over the same time period (A–C; n = 30–31). (D) Body weight progression of GIPR^ΔGAT^ mice over 8 weeks of HFD feeding with (E) subsequent body composition measurement. (F) Food intake measurements for the GIPR^ΔGAT^ mice over the same time period (D–F; n = 30). (G) Locomotor activity, (H) respiratory exchange ratio, (I) and energy expenditure measurements from GIPR^ΔGAT^ mice placed in metabolic cages (G–I; n = 11). (J) Body weight progression of GIPR^ΔGLUT^ mice over 8 weeks of HFD feeding with (K) subsequent body composition measurements. (L) Food intake of the GIPR^ΔGAT^ mice over the same time period (J–L; n = 20–21). Data in A, C, D, F, J, and L were analyzed via a repeated measures 2-way ANOVA with Bonferroni's multiple comparisons test. Data in B, E, and K were analyzed with a Student's two-sided, two tailed t-test. Data in G and H were analyzed with a 2-way ANOVA. Data in I was analyzed with ANCOVA using total body mass as a covariate. Data are displayed as mean ± SEM. ∗p < 0.05; ∗∗p < 0.01; ∗∗∗p < 0.001; ∗∗∗∗p < 0.0001.

The global GIPR KO (GIPR^KO^) was protective against HFD-induced obesity as has been previously reported [33]. Body weight, lean mass, and fat mass between genotypes were indistinguishable prior to HFD (Supplemental 2A, n = 30–31) but diverged after the addition of HFD (Figure 1A–B, n = 30–31). After 8 weeks of HFD, KO mice had lower total mass, lean mass, and fat mass than their WT littermates (Figure 1B, n = 30–31). GIPR^KO^ mice consumed less food than WT mice both cumulatively (Figure 1C, n = 30–31) and daily (Supplemental 2B: n = 30–31). There were no genotype differences in a fast/refeed assay (Supplemental 2C: n = 12).

The GABAergic neuron GIPR KO (GIPR^ΔGAT^) was also protective against HFD-induced obesity. Body weight, lean mass, and fat mass between genotypes were indistinguishable before HFD (Supplemental 2D, n = 30) but diverged after the addition of HFD (Figure 1D–E; n = 30). After 8 weeks of HFD, GIPR^ΔGAT^ mice had lower total, lean, and fat mass than their WT littermates (Figure 1E; n = 30). GIPR^ΔGAT^ mice consumed less food than WT mice both cumulatively (Figure 1F, n = 30) and daily (Supplemental 2E, n = 30). When placed into metabolic chambers (WT n = 11; KO n = 11), WT and GIPR^ΔGAT^ mice performed identically in measurements of locomotor activity (Figure 1G), respiratory exchange ratio (Figure 1H), and energy expenditure as analyzed using ANCOVA with total mass as a covariate since mice primarily differed in fat mass [46] (Figure 1I). There were no genotype differences in a fast/refeed assay (Supplemental 2F: n = 11–12).

Meal pattern data was generated by the TSE metabolic cages for the same cohort of GIPR^ΔGAT^ mice before and after HFD feeding. Meal pattern parameters were analyzed both by averaging per mouse as well as by pooling from all mice in a genotype. There were no genotype differences seen in any measurement for both the lean and DIO mice (Supplemental Figure 3 A-P, n = 10–11). Since there were no genotype differences, mice were pooled, and the parameters were compared before and after HFD feeding. DIO HFD-fed mice had a higher average meal count, smaller average meal size, and shorter average meal duration than when they were lean and chow-fed with no differences in average inter-meal interval (Supplemental Figure 3 Q-T, n = 21).

The glutamatergic neuron GIPR KO (GIPR^ΔGLUT^) was not protective against HFD-induced obesity. Body weight, lean mass, and fat mass between genotypes were indistinguishable before HFD (Supplemental 2G, n = 20–21) and remained this way after the addition of HFD (Figure 1J-K; n = 20–21). After 8 weeks of HFD, GIPR^ΔGLUT^ mice had indistinguishable total mass, lean mass, and fat mass from their WT littermates (Figure 1K; n = 20–21). WT and GIPR^ΔGLUT^ mice consumed the same amount of food both cumulatively (Figure 1L, n = 20–21) and daily (Supplemental 2H, n = 20–21). Since GIPR^ΔGLUT^ mice had no discernible phenotype, they were not placed into metabolic cages for further analysis. There were no genotype differences in a fast/refeed assay (Supplemental 2I: n = 20–21).

### 3.2 Body weight and food intake responses to chronic administration of incretin drugs

**Figure 2:**
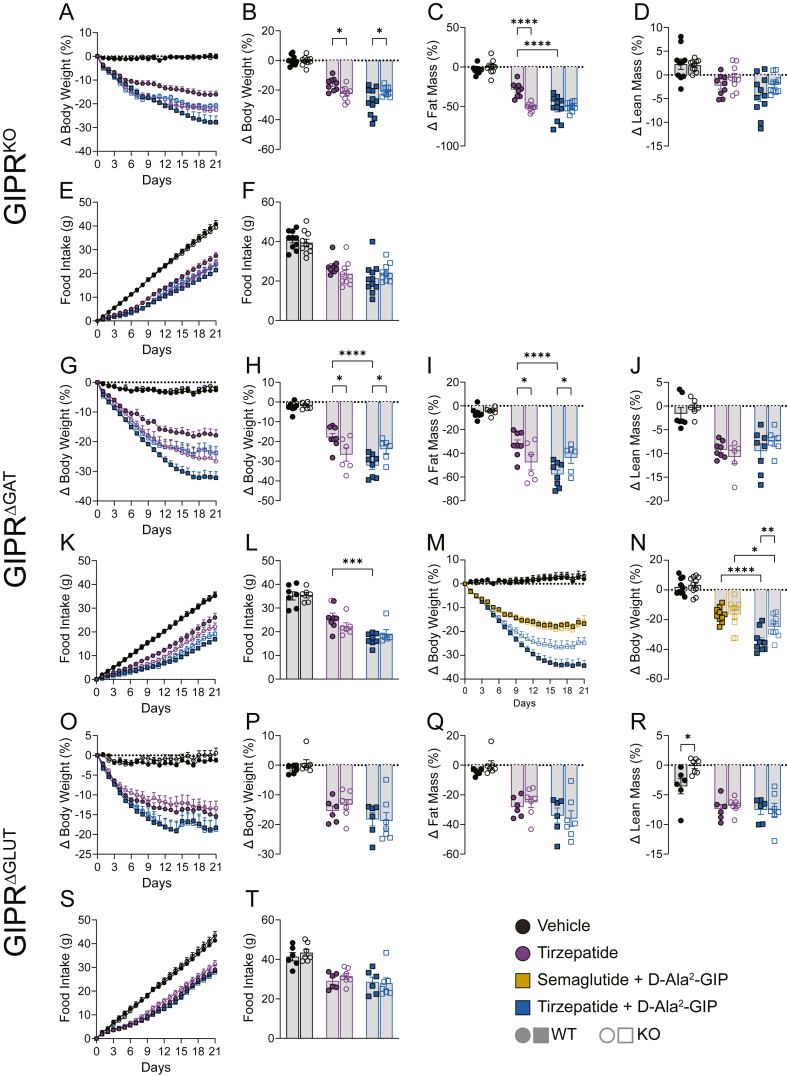
**GIPR^KO^ and GIPR^ΔGAT^ enhance GLP-1R agonism and block the enhanced efficacy of DIA**. (A–B) Body weight, (C) fat mass, (D) and lean mass change in GIPR^KO^ mice in response to 21 days of daily incretin drugs (n = 9–11). (E–F) Food intake responses to incretin drugs in the same cohort of GIPR^KO^ mice (n = 9–11). (G–H) Body weight, (I) fat mass and (J) lean mass change in GIPR^ΔGAT^ mice in response to 21 days of daily incretin drugs (n = 6–8). (K–L) Food intake responses to incretin drugs in during the same period (n = 6–8). (M–N) Body weight responses to incretin drugs in a second cohort of GIPR^ΔGAT^ mice (n = 10). (O–P) Body weight, (Q) fat mass, (R) and lean mass change in GIPR^ΔGLUT^ mice in response to 21 days of daily incretin drugs (n = 6–7). (S–T) Food intake responses during the same period (n = 6–7). Data in B, C, D, F, H, I, J, L, N, P, Q, R, and T were analyzed using a 2-way ANOVA. Data are displayed as mean ± SEM. ∗p < 0.05; ∗∗p < 0.01; ∗∗∗p < 0.001; ∗∗∗∗p < 0.0001.

After 21 days of daily dosing with the indicated drugs, GIPR^KO^ mice lost more body weight and fat mass in response to tirzepatide compared to WTs (Figure 2B–C, n = 9–11). Additionally, the enhanced weight loss efficacy of the tirzepatide plus GIPR agonist treatment was prevented by global deletion of GIPR, confirming that the effect was GIPR-mediated (Figure 2B, n = 9–11). There were no genotype differences observed on lean mass change (Figure 2D, n = 59) or food intake (Figure 2E–F, n = 9–11).

After 21 days of chronic dosing, GIPR^ΔGAT^ mice lost more weight and fat mass when given tirzepatide compared to their WT littermates (Figure 2G–H, n = 6–8). Combining tirzepatide with GIPR agonism caused a greater degree of weight loss and fat mass loss than tirzepatide alone. This effect was prevented in GIPR^ΔGAT^ mice (Figure 2H–I n = 6–8), suggesting that GIPR signaling in these neurons is required for the enhanced weight loss efficacy of DIA therapy over GLP-1R agonism alone. Changes in lean mass were indistinguishable between WT and GIPR^ΔGAT^ mice in drug groups (Figure 2J, n = 6–8). The tirzepatide + GIPR agonist group was superior to tirzepatide alone in reducing food intake but there were no genotype effects (Figure 2K–L, n = 6–8). In a second cohort of GIPR^ΔGAT^ mice given either semaglutide + D-Ala^2^-GIP or tirzepatide + D-Ala^2^-GIP, only the tirzepatide group showed a genotype difference (Figure 2N, n = 10). This could indicate that either the combination of tirzepatide's modest GIPR activity with another GIPR agonist was sufficient to alter body weight in these mice or that tirzepatide's unique pharmacology at the GLP-1R allowed for better interaction with GIPR agonism [47].

After 21 days of chronic dosing, GIPR^ΔGLUT^ mice performed identically to WT littermates in terms of weight loss, fat mass, and food intake responses to each drug group (Figure 2P–Q and T, n = 6–7). Interestingly, GIPR^ΔGLUT^ mice lost less lean mass in only the vehicle group (Figure 2R, n = 6–7). Combined with the findings from Figure 1, this suggests that glutamatergic GIPR neurons are not involved in either physiological energy balance or response to weight-loss medications.

**Figure 3:**
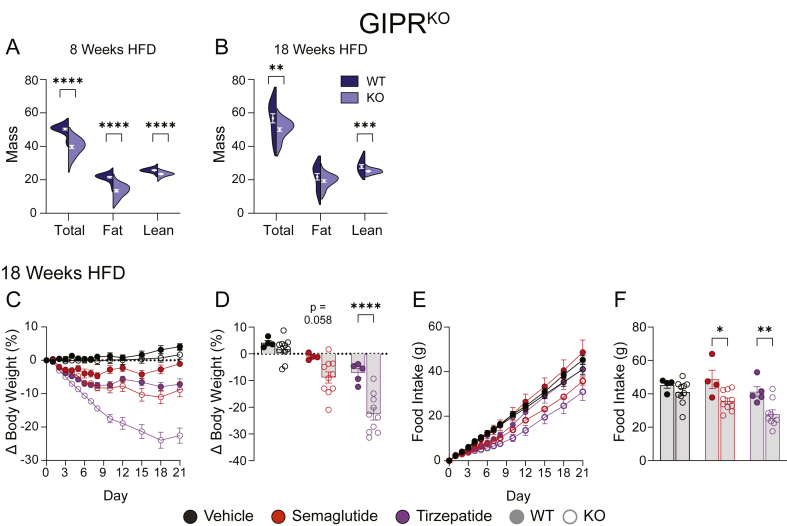
**GLP-1R agonism is enhanced in whole-body GIPR^KO^ mice**. (A) Body composition of the cohort of GIPR^KO^ mice from Figure 2 prior to incretin drug administration (n = 29–31). (B) Body composition of the cohort of GIPR^KO^ mice used in C–F prior to incretin drug administration (n = 13–30). (C–D) Body weight and (E–F) food intake responses of GIPR^KO^ mice in response to 21 days of daily incretin drugs (n = 4–10). Data in A and B were analyzed via Student's two-sided, two tailed t-test. Data in D and F were analyzed using a 2-way ANOVA. Data are displayed as mean ± SEM. ∗p < 0.05; ∗∗p < 0.01; ∗∗∗p < 0.001; ∗∗∗∗p < 0.0001.

The fat mass of the GIPR^KO^ mice in Figure 2 was relatively low compared to WT counterparts due to the protective effect of the knockout (Figure 3A, n = 6–7). We theorized that mice with a greater amount of fat mass to lose might show an enhanced response to drug administration, so we generated a second cohort of GIPR^KO^ mice that was given HFD for a longer 18-week period. This resulted in GIPR^KO^ mice that had statistically indistinguishable fat mass (Figure 3B, n = 13–30). After 21 days of chronic dosing in this cohort, KO mice trended towards more weight loss in response to semaglutide (p = 0.058) and experienced substantially more weight loss in response to tirzepatide compared to WT littermates (Figure 3D, n = 4–10). Similarly, GIPR^KO^ mice consumed less food in response to both semaglutide and tirzepatide compared to WT littermates (Figure 3F, n = 4–10).

### 3.3 Conditioned taste aversion

**Figure 4:**
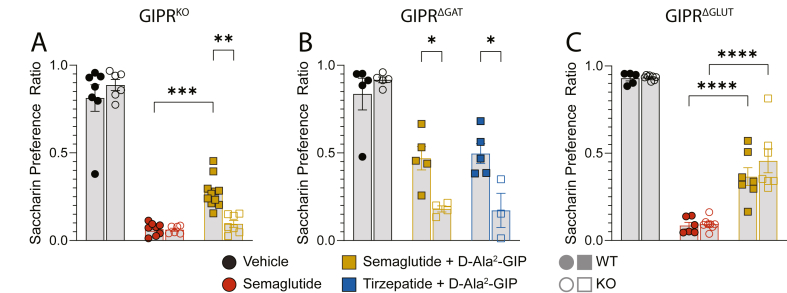
**GIPR^KO^ and GIPR^ΔGAT^ prevent the anti-aversive actions of GIPR agonism**. (A) 24 h measurement of saccharin preference ratio of GIPR^KO^ mice in a conditioned taste aversion assay (n = 7–11). (B) 24 h measurement of saccharin preference ratio of GIPR^ΔGAT^ mice in a conditioned taste aversion assay (n = 3–5). (C) 24 h measurement of saccharin preference ratio of GIPR^ΔGLUT^ mice in a conditioned taste aversion assay (n = 5–8). All data was analyzed via 2-way ANOVA. Data are displayed as mean ± SEM. ∗p < 0.05; ∗∗p < 0.01; ∗∗∗p < 0.001; ∗∗∗∗p < 0.0001.

The reduction in GLP-1RA-mediated aversion by GIPR agonism was blocked in GIPR^KO^ (Figure 4A, n = 6–11). Similarly, lean male mice GIPR^ΔGAT^ showed more aversion in response to DIA drug combinations than their WT counterparts (Figure 4B, n = 3–5). Male GIPR^ΔGLUT^ mice showed no genotype differences (Figure 4C, n = 6–8). In lean females, GIPR^KO^ mice responded with more aversion in the DIA group than WT counterparts with no genotype difference to GLP-1R agonism alone (Supplemental Figure 4 A, n = 4–7). The reduction in aversion caused by GIPR agonism was blocked in lean female GIPR^ΔGAT^ mice (Supplemental Figure 4 B, n = 3–4). In contrast to the male data, GIPR^ΔGLUT^ lean females showed less aversion in response to DIA than did their WT littermates (Supplemental Figure 4C, n = 7). This may indicate a sex difference in the interaction of glutamatergic GIPR neurons and aversion.

## 4 Discussion

DIAs are potent therapeutic agents with the potential to produce large and sustained reductions in body weight. Identifying the neural populations that they target is an important research goal. The need to do this is made even more urgent given the confusing findings that drugs that are GLP-1R agonists and GIP antagonists can also produce significant weight loss in both pre-clinical models and humans [15,48]. Our work using large cohorts of mice complements the work of Liskiewicz et al., who found reduced GIPR signaling in GABAergic neurons replicates the resistance to weight gain on an HFD of whole-body GIPR knockout mice [49]. We have further extended these findings to indicate that there is very little effect of reducing GIPR signaling in excitatory glutamatergic neurons on food intake and body weight.

This has a couple of important implications. First, reductions in GIPR signaling in GABAergic neurons contribute to normal energy balance regulation but only in the context of an obesogenic food environment, a pattern seen previously in whole-body GIPR knockouts [33]. This also fits with recent data that point to CNS GIPR signaling as the key component of how GIP is linked to the regulation of energy balance rather than actions in peripheral organs, such as adipose tissue [16,49]. In our hands, the reduced weight gain in GIPR^KO^ and GIPR^ΔGAT^, but not GIPR^ΔGLUT^, mice is associated primarily with reduced consumption of the HFD rather than alterations in energy expenditure or nutrient handling as has been suggested by other studies [33,50]. However, it is difficult to dissociate differences in food intake caused by smaller body size versus those caused by a direct effect on food intake of GIPR KO itself. Indeed, the genotype differences in food intake disappear when analyzed via a linear mixed model using body weight as a covariate at each time point (not shown). To minimize thermostress, all mice in this study were housed at 30 °C, allowing the mice to better emulate human responses to diet and drug administration. The body weight effects in Figure 1 match published data for the GIPR^KO^ and GIPR^ΔGAT^ knockouts at room temperature [33,49] but two previous studies found that thermoneutrality negates the protective effect of both GLP-1R and GIPR knockouts on body weight [51,52]. The cause of this discrepancy is unclear. The second implication is that genetic manipulations to reduce GIPR signaling in the appropriate inhibitory GABAergic neuronal populations are associated with lower weight on an HFD, suggesting that pharmacological GIPR antagonism in this population may be beneficial.

Due to tirzepatide's low affinity for the mouse GIPR, we combined a low dose of tirzepatide with a separate GIPR agonist to emulate the human response to DIA [38,39]. The alternate option, using a very high tirzepatide dose as in [53], presents its own issues for interpretation. Considering that chronic administration of 2 nmol/kg tirzepatide caused >20% mean body weight reduction, a 30 nmol/kg dose would likely reach a saturation point where any GIPR-mediated differences in body weight would disappear due to maximal GLP-1R engagement. We would expect a similar oversaturation effect in a CTA experiment where no amount of GIPR agonism would be sufficient to overcome the extreme aversive response from high dose GLP-1R agonism.

The finding that the additional weight loss benefit of adding GIPR activity to a GLP-1R agonist is mediated by GIPR signaling in inhibitory GABAergic neurons [49], rather than in excitatory glutamatergic neurons has implications to better understand the actions of DIAs. First, these data provide evidence that GIPR signaling in only GABAergic neurons is crucial to the response of such DIAs for reducing food intake and body weight even though GIPR is found in the periphery as well as non-neuronal cell types in the CNS [26,54]. Second, these data strongly indicate that the GLP-1 and GIP components of these DIAs act on separate populations of neurons that ultimately act together in coordination to further reduce food intake than either monoagonist. Unlike what was found here for GIPR, GLP-1R agonists require GLP-1 signaling on glutamatergic but not GABAergic neurons [55], though this finding is not universally agreed upon [56]. This means that the synergistic actions of GLP-1R and GIPR agonists in the context of a DIA are distinct from what happens on pancreatic β-cells which express both GLP-1 and GIP receptors, engaging common intracellular signaling pathways [57,58]. Rather, the current data would support a model where DIAs act on separate populations of neurons that likely either directly communicate with each other or have common downstream targets that regulate food intake. This is supported by the findings that GLP-1R agonism and GIPR agonism activate separate brainstem populations [19] and that the two receptors are typically found in different neuron types [28,29]. A wide range of data raise the possibility that both of these populations of neurons may reside within the area postrema (AP) that sits outside the blood–brain barrier and consequently are ideally situated to sense circulating levels of GIP [27,55,59].

The final implication of these data is that loss of GIPR signaling in GABAergic but not glutamatergic neurons enhances the ability of a GLP-1R monoagonist to reduce food intake and body weight. It seems counterintuitive that tirzepatide's actions are enhanced in both whole-body and GABAergic GIPR knockouts. After all, as it is a DIA, such data imply that losing the GIPR agonist component increases rather than decreases its effectiveness. However, in the context of mouse studies, the actions of tirzepatide are probably best thought of as reflecting only the GLP-1 component of the molecule because it is a relatively poor agonist for the mouse GIP receptor [38,39]. In light of this, we interpret the findings from both Figure 2, Figure 3 to mean that loss of GIPR signaling can enhance the weight loss from GLP-1R agonism. To wit, this finding is not without precedent as similar results have been seen before with Mroz et al. finding that a GLP-1 analog peptide caused substantially more weight loss in whole-body GIPR KO mice compared to WT counterparts [38]. Furthermore, pharmacological evidence makes it clear that an antibody that acts as a GIPR antagonist can enhance the weight loss effects of GLP-1R agonism [30]. The current data imply that such increases in the effectiveness of a GLP-1R agonist can be achieved by genetic approaches to reducing GIPR signaling and that the key targets are GABAergic neurons that express the GIPR.

One hypothesis to explain the apparent contradiction that both GIPR agonism and antagonism can enhance the efficacy of a GLP-1R agonist is that the key actions of agonists and antagonists are on separate populations of receptors [36,60]. This is a logical hypothesis particularly given that the clinically effective GLP-1R agonist/GIPR antagonist is an antibody that may be distributed very differently than a peptide and thereby preferentially act upon separate populations of GIPR. However, our data make a strong case that the increased weight loss which can accompany either a GIPR agonist or antagonist depends on GIPR signaling in GABAergic neurons. It remains possible that the peptide and antibody may interact with different populations of GIPR in different anatomical locations within the CNS, since the antibody protein is considerably larger than either tirzepatide or D-Ala^2^-GIP peptides. However, multiple recent studies demonstrate that GIPR agonists locate primarily to circumventricular organs outside the blood–brain barrier such as the AP [26,49].

The AP is also an ideal candidate for a brain region that would mediate the effects of a large antibody that is highly unlikely to gain access to other regions of the brain protected by a normal blood–brain barrier. Furthermore, the GABAergic GIPR neurons in the AP directly inhibit aversive and anorectic AP neurons [20,28], suggesting that GIPR antagonism (or functional antagonism via chronic GIPR stimulation [37]) of these neurons may improve the actions of GLP-1R agonists and be therapeutically beneficial as compared to either GLP-1R agonists or DIAs. Consequently, we conclude that the key GIPR populations for both a DIA and a GlPR antagonist antibody substantially overlap. This study's findings beg the question of why a GIPR KO in GABAergic neurons would produce this effect in the first place since it is unclear if any basal GIPR tone exists in the brain due to GIP's rapid degradation by DPP-4 [61]. Importantly, GIPR has a much greater constitutive activity than does GLP-1R [62]. Thus, one possible explanation for the effects is an innate ligand-independent GIPR tone in GABAergic AP neurons, the removal of which disinhibits local AP anorectic neurons.

If GIPR antagonism decreases body weight by increasing the actions of anorectic AP neurons, it could also increase nausea symptoms. While it is true that a GLP-1R agonist/GIPR antagonist caused nausea or vomiting in a much higher proportion of patients than did semaglutide at approximately the same amount of weight loss, this study was too small to make definitive conclusions about the side effect profile [14,30]. Notably, GIPR antagonists given to humans or cynomolgus monkeys produced no side effects, suggesting that GIPR antagonism does not produce nausea [63,64]. As synaptic disinhibition in the AP may not present identically to direct activation of aversive neurons, this finding does not necessarily preclude the model proposed above.

The current study demonstrates the requirement for GIPR in GABAergic neurons for GIPR-mediated reduction in aversion as well as the enhanced weight loss efficacy of DIAs. Furthermore, we show that the removal of GIPR from GABAergic neurons is protective against HFD and enhances the effects of both semaglutide and tirzepatide in mice. Together these data indicate that GABAergic GIPR neurons represent multiple populations of metabolically active neurons with distinct and opposing effects. Future identification of the exact neurons and/or neuronal circuitries responsible for each effect will tease out a more precise targeting of future pharmacotherapies for body weight loss with less aversive effects.

## Funding sources

This work was supported by 10.13039/100000002National Institutes of Health grants to R.J.S. (grant numbers P30DK089503 and R01DK133140) and by 10.13039/100004325AstraZeneca.

## CRediT authorship contribution statement

**Jordan Wean:** Writing – original draft, Visualization, Software, Investigation, Formal analysis, Conceptualization. **Allison Ho Kowalsky:** Writing – review & editing, Investigation, Formal analysis, Conceptualization. **Rhianna Laker:** Conceptualization. **Sarah Will:** Conceptualization. **Daniel J. Drucker:** Writing – review & editing, Resources. **Christopher J. Rhodes:** Writing – review & editing, Supervision, Conceptualization. **Randy J. Seeley:** Writing – review & editing, Supervision, Funding acquisition, Conceptualization.

## Declaration of competing interest

R.J.S. has received research support from Novo Nordisk, Fractyl, Astra Zeneca, Congruence Therapeutics, Eli Lilly, Bullfrog AI, Glyscend Therapeutics and Amgen. RJS has served as a paid consultant for Novo Nordisk, Eli Lilly, CinRx, Fractyl, Structure Therapeutics, Crinetics, Amgen and Congruence Therapeutics. R.J.S. has equity in Bullfrog AI and Rewind. R.L., S.W., and C.R. are employees of AstraZeneca. DJD has received remuneration for consulting or speaking from Amgen, AstraZeneca, Boerhinger Ingelheim Kallyope, Merck, Novo Nordisk, Pfizer Inc and Zeleand Pharma and Mt. Sinai Hospital receives investigator-initiated grant support for studies in the Drucker lab from Amgen, 10.13039/501100004191Novo Nordisk, 10.13039/100004319Pfizer and Zealand Pharma Inc.

## Data Availability

Data will be made available on request.

## Abbreviations

AP: Area postrema
BMI: Body Mass Index
CNS: Central nervous system
CTA: Conditioned taste aversion
DIA: Dual incretin agonist
DIO: Diet-induced obese
GABA: Gamma-aminobutyric acid
GIP: Glucose-dependent insulinotropic polypeptide
GIPRKO: Whole-body GIPR knockout
GIPR^ΔGAT^: GABAergic neuron GIPR knockout
GIPR^ΔGLUT^: Glutamatergic neuron GIPR knockout
GLP-1: Glucagon-like peptide-1
HFD: High-fat diet

## Appendix A Supplementary data

Multimedia component 1 D-Ala^2^-GIP characterization and dosage gradients. (A) qPCR of GIPR in white adipose tissue of GIPR^KO^ mice (n = 4–5). Food intake measurements after a single dose of D-Ala^2^-GIP at (B) 30 nmol/kg or (C) 150 nmol/kg (B; n = 5–6, C; n = 5). (D) Conditioned taste aversion assay comparing D-Ala^2^-GIP to semaglutide and tirzepatide (n = 5). (E) Dosage gradient for tirzepatide in a conditioned taste aversion assay (n = 5–11). (F) Dosage gradient for tirzepatide in a short-term food intake assay (n = 4–30). (G) Dosage gradient for semaglutide in a short-term food intake assay (n = 5–30). Data in C–F was analyzed via 1-way ANOVA. Data are displayed as mean ± SEM. # indicates significance versus vehicle. #p < 0.05; ##p < 0.01; ###p < 0.001; ∗∗∗∗p < 0.0001. Multimedia component 1

Multimedia component 2 Phenotypic characterization of GIPR KO models. (A) Body composition measurements of GIPR^KO^ mice before HFD feeding. (B) Daily food intake for GIPR^KO^ mice during HFD feeding (A–B; n = 30–31). (C) Fast/refeed assay for GIPR^KO^ mice (n = 12). (D) Body composition measurements of GIPR^ΔGAT^ mice before HFD feeding. (E) Daily food intake for GIPR^ΔGAT^ mice during HFD feeding (D–E; n = 30). (F) Fast/refeed assay for GIPR^ΔGAT^ mice (n = 11–12). (G) Body composition measurements of GIPR^ΔGLUT^ mice before HFD feeding. (H) Daily food intake for GIPR^ΔGLUT^ mice during HFD feeding (G–H; n = 20–21). (I) Fast/refeed assay for GIPR^ΔGLUT^ mice (n = 12). Data in A, D, G were analyzed via two-tailed t-test. Data in B, C, E, F, H, and I were analyzed via a repeated measures 2-way ANOVA. ∗p < 0.05; ∗∗p < 0.01; ∗∗∗p < 0.001; ∗∗∗∗p < 0.0001. Multimedia component 2

Multimedia component 3 Meal pattern analysis for GIPR^ΔGAT^ mice before and after DIO. (A) Meal patterns for three days in lean GIPR^ΔGAT^ mice. Each horizontal gray line represents a mouse. (B) Average meal count, (C) average meal size, (D) average meal duration and (E) average inter-meal interval per mouse (A–E; n = 10–11). (F) Pooled meal size, (G) meal duration, (H) and inter-meal interval. (I) Meal patterns for 3 days in DIO GIPR^ΔGAT^ mice. These are the same mice from A–H. (J) Average meal count, (K) average meal size, (L) average meal duration and (M) average inter-meal interval per mouse (I–M; n = 10–11). (N) Pooled meal size, (O) meal duration, (P) and inter-meal interval. (Q) Paired analyses of average meal count, (R) average meal size (S) average meal duration, (T) and average inter-meal interval of mice before and after induction of DIO via HFD feeding (n = 21). Data from B–H and J–P were analyzed via 2-way ANOVA. Data in Q, R, and T were analyzed via paired Student's t-test, and the data in S was analyzed via a Wilcoxon matched-pairs signed rank test. Data in B–H and J–P were displayed as a standard box and whisker plot: minimum, lower quartile, median, upper quartile, and maximum. ∗p < 0.05; ∗∗p < 0.01; ∗∗∗p < 0.001; ∗∗∗∗p < 0.0001. Multimedia component 3

Multimedia component 4 Female conditioned taste aversion. (A) 24 h. measurement of saccharin preference ratio of female GIPR^KO^ mice in a conditioned taste aversion assay (n = 4–7). (B) 24 h. measurement of saccharin preference ratio of female GIPR^ΔGAT^ mice in a conditioned taste aversion assay (n = 3–4). (C) 24 h. measurement of saccharin preference ratio of female GIPR^ΔGLUT^ mice in a conditioned taste aversion assay (n = 7). All data was analyzed via 2-way ANOVA. Data are displayed as mean ± SEM. ∗p < 0.05; ∗∗p < 0.01; ∗∗∗p < 0.001; ∗∗∗∗p < 0.0001. Multimedia component 4

## References

[1] World Obesity Federation (2024).

[2] Global B.M.I.M.C., Di Angelantonio E., Bhupathiraju Sh N., Wormser D., Gao P., Kaptoge S. (2016). Body-mass index and all-cause mortality: individual-participant-data meta-analysis of 239 prospective studies in four continents. Lancet.

[3] Powell-Wiley T.M., Poirier P., Burke L.E., Despres J.P., Gordon-Larsen P., Lavie C.J. (2021). Obesity and cardiovascular disease: a scientific statement from the American Heart association. Circulation.

[4] Kraschnewski J.L., Boan J., Esposito J., Sherwood N.E., Lehman E.B., Kephart D.K. (2010). Long-term weight loss maintenance in the United States. Int J Obes.

[5] Flore G., Preti A., Carta M.G., Deledda A., Fosci M., Nardi A.E. (2022). Weight maintenance after dietary weight loss: systematic review and meta-analysis on the effectiveness of behavioural intensive intervention. Nutrients.

[6] Hall K.D., Kahan S. (2018). Maintenance of lost weight and long-term management of obesity. Med Clin North Am.

[7] Aronne L.J., Hall K.D., J M.J., Leibel R.L., Lowe M.R., Rosenbaum M. (2021). Describing the weight-reduced state: physiology, behavior, and interventions. Obesity (Silver Spring).

[8] Berthoud H.R., Seeley R.J., Roberts S.B. (2021). Physiology of energy intake in the weight-reduced state. Obesity (Silver Spring).

[9] Laiteerapong N., Huang E.S. (2010). The public health implications of the cost-effectiveness of bariatric surgery for diabetes. Diabetes Care.

[10] Müller T.D., Blüher M., Tschöp M.H., Dimarchi R.D. (2021). Anti-obesity drug discovery: advances and challenges. Nat Rev Drug Discov.

[11] Muller T.D., Clemmensen C., Finan B., DiMarchi R.D., Tschop M.H. (2018). Anti-obesity therapy: from rainbow pills to polyagonists. Pharmacol Rev.

[12] Müller T.D., Finan B., Bloom S.R., D'Alessio D., Drucker D.J., Flatt P.R. (2019). Glucagon-like peptide 1 (GLP-1). Mol Metab.

[13] Campbell J.E., Drucker D.J. (2013). Pharmacology, physiology, and mechanisms of incretin hormone action. Cell Metab.

[14] Wilding J.P.H., Batterham R.L., Calanna S., Davies M., Van Gaal L.F., Lingvay I. (2021). Once-weekly semaglutide in adults with overweight or obesity. N Engl J Med.

[15] Jastreboff A.M., Aronne L.J., Ahmad N.N., Wharton S., Connery L., Alves B. (2022). Tirzepatide once weekly for the treatment of obesity. N Engl J Med.

[16] Zhang Q., Delessa C.T., Augustin R., Bakhti M., Colldén G., Drucker D.J. (2021). The glucose-dependent insulinotropic polypeptide (GIP) regulates body weight and food intake via CNS-GIPR signaling. Cell Metab.

[17] Samms R.J., Coghlan M.P., Sloop K.W. (2020). How may GIP enhance the therapeutic efficacy of GLP-1?. Trends Endocrinol Metab.

[18] Borner T., Geisler C.E., Fortin S.M., Cosgrove R., Alsina-Fernandez J., Dogra M. (2021). GIP receptor agonism attenuates GLP-1 receptor agonist induced nausea and emesis in preclinical models. Diabetes.

[19] Costa A., Ai M., Nunn N., Culotta I., Hunter J., Boudjadja M.B. (2022). Anorectic and aversive effects of GLP-1 receptor agonism are mediated by brainstem cholecystokinin neurons, and modulated by GIP receptor activation. Mol Metab.

[20] Zhang C., Vincelette L.K., Reimann F., Liberles S.D. (2022). A brainstem circuit for nausea suppression. Cell Rep.

[21] Samms R.J., Cosgrove R., Snider B.M., Furber E.C., Droz B.A., Briere D.A. (2022). GIPR agonism inhibits PYY-induced nausea-like behavior. Diabetes.

[22] Rosenstock J., Wysham C., Frias J.P., Kaneko S., Lee C.J., Fernandez Lando L. (2021). Efficacy and safety of a novel dual GIP and GLP-1 receptor agonist tirzepatide in patients with type 2 diabetes (SURPASS-1): a double-blind, randomised, phase 3 trial. Lancet.

[23] Adams J.M., Pei H., Sandoval D.A., Seeley R.J., Chang R.B., Liberles S.D. (2018). Liraglutide modulates appetite and body weight through glucagon-like peptide 1 receptor–expressing glutamatergic neurons. Diabetes.

[24] Sisley S., Gutierrez-Aguilar R., Scott M., D'Alessio D.A., Sandoval D.A., Seeley R.J. (2014). Neuronal GLP1R mediates liraglutide's anorectic but not glucose-lowering effect. J Clin Invest.

[25] Secher A., Jelsing J., Baquero A.F., Hecksher-Sørensen J., Cowley M.A., Dalbøge L.S. (2014). The arcuate nucleus mediates GLP-1 receptor agonist liraglutide-dependent weight loss. J Clin Invest.

[26] Adriaenssens A., Broichhagen J., de Bray A., Ast J., Hasib A., Jones B. (2023). Hypothalamic and brainstem glucose-dependent insulinotropic polypeptide receptor neurons employ distinct mechanisms to affect feeding. JCI Insight.

[27] Ludwig M.Q., Cheng W., Gordian D., Lee J., Paulsen S.J., Hansen S.N. (2021). A genetic map of the mouse dorsal vagal complex and its role in obesity. Nat Metab.

[28] Zhang C., Kaye J.A., Cai Z., Wang Y., Prescott S.L., Liberles S.D. (2021). Area postrema cell types that mediate nausea-associated behaviors. Neuron.

[29] Adriaenssens A.E., Biggs E.K., Darwish T., Tadross J., Sukthankar T., Girish M. (2019). Glucose-dependent insulinotropic polypeptide receptor-expressing cells in the hypothalamus regulate food intake. Cell Metabol.

[30] Veniant M.M., Lu S.C., Atangan L., Komorowski R., Stanislaus S., Cheng Y. (2024). A GIPR antagonist conjugated to GLP-1 analogues promotes weight loss with improved metabolic parameters in preclinical and phase 1 settings. Nat Metab.

[31] Speliotes E.K., Willer C.J., Berndt S.I., Monda K.L., Thorleifsson G., Jackson A.U. (2010). Association analyses of 249,796 individuals reveal 18 new loci associated with body mass index. Nat Genet.

[32] Styrkarsdottir U., Tragante V., Stefansdottir L., Thorleifsson G., Oddsson A., Sørensen E. (2024). Obesity variants in the *GIPR* gene are not associated with risk of fracture or bone mineral density. J Clin Endocrinol Metab.

[33] Miyawaki K., Yamada Y., Ban N., Ihara Y., Tsukiyama K., Zhou H. (2002). Inhibition of gastric inhibitory polypeptide signaling prevents obesity. Nat Med.

[34] Althage M.C., Ford E.L., Wang S., Tso P., Polonsky K.S., Wice B.M. (2008). Targeted ablation of glucose-dependent insulinotropic polypeptide-producing cells in transgenic mice reduces obesity and insulin resistance induced by a high fat diet. J Biol Chem.

[35] Nasteska D., Harada N., Suzuki K., Yamane S., Hamasaki A., Joo E. (2014). Chronic reduction of GIP secretion alleviates obesity and insulin resistance under high-fat diet conditions. Diabetes.

[36] Svendsen B., Capozzi M.E., Nui J., Hannou S.A., Finan B., Naylor J. (2020). Pharmacological antagonism of the incretin system protects against diet-induced obesity. Mol Metab.

[37] Killion E.A., Chen M., Falsey J.R., Sivits G., Hager T., Atangan L. (2020). Chronic glucose-dependent insulinotropic polypeptide receptor (GIPR) agonism desensitizes adipocyte GIPR activity mimicking functional GIPR antagonism. Nat Commun.

[38] Mroz P.A., Finan B., Gelfanov V., Yang B., Tschop M.H., DiMarchi R.D. (2019). Optimized GIP analogs promote body weight lowering in mice through GIPR agonism not antagonism. Mol Metab.

[39] Samms R.J., Zhang G., He W., Ilkayeva O., Droz B.A., Bauer S.M. (2022). Tirzepatide induces a thermogenic-like amino acid signature in brown adipose tissue. Mol Metab.

[40] Concordet J.-P., Haeussler M. (2018). CRISPOR: intuitive guide selection for CRISPR/Cas9 genome editing experiments and screens. Nucleic Acids Res.

[41] Campbell J.E., Ussher J.R., Mulvihill E.E., Kolic J., Baggio L.L., Cao X. (2016). TCF1 links GIPR signaling to the control of beta cell function and survival. Nat Med.

[42] Lamont B.J., Drucker D.J. (2008). Differential antidiabetic efficacy of incretin agonists versus DPP-4 inhibition in high fat–fed mice. Diabetes.

[43] Mina A.I., Leclair R.A., Leclair K.B., Cohen D.E., Lantier L., Banks A.S. (2018). CalR: a web-based analysis tool for indirect calorimetry experiments. Cell Metabol.

[44] Waskom M. (2021). seaborn: statistical data visualization. J Open Source Softw.

[45] Wickham H. (2016).

[46] Virtue S., Lelliott C.J., Vidal-Puig A. (2021). What is the most appropriate covariate in ANCOVA when analysing metabolic rate?. Nat Metab.

[47] Willard F.S., Douros J.D., Gabe M.B., Showalter A.D., Wainscott D.B., Suter T.M. (2020). Tirzepatide is an imbalanced and biased dual GIP and GLP-1 receptor agonist. JCI Insight.

[48] Coskun T., Sloop K.W., Loghin C., Alsina-Fernandez J., Urva S., Bokvist K.B. (2018). LY3298176, a novel dual GIP and GLP-1 receptor agonist for the treatment of type 2 diabetes mellitus: from discovery to clinical proof of concept. Mol Metab.

[49] Liskiewicz A., Khalil A., Liskiewicz D., Novikoff A., Grandl G., Maity-Kumar G. (2023). Glucose-dependent insulinotropic polypeptide regulates body weight and food intake via GABAergic neurons in mice. Nat Metab.

[50] Hansotia T., Maida A., Flock G., Yamada Y., Tsukiyama K., Seino Y. (2007). Extrapancreatic incretin receptors modulate glucose homeostasis, body weight, and energy expenditure. J Clin Invest.

[51] Furber E.C., Hyatt K., Collins K., Yu X., Droz B.A., Holland A. (2024). GIPR agonism enhances TZD-induced insulin sensitivity in obese IR mice. Diabetes.

[52] Samms R.J., Christe M.E., Collins K.A., Pirro V., Droz B.A., Holland A.K. (2021). GIPR agonism mediates weight-independent insulin sensitization by tirzepatide in obese mice. J Clin Invest.

[53] El K., Douros J.D., Willard F.S., Novikoff A., Sargsyan A., Perez-Tilve D. (2023). The incretin co-agonist tirzepatide requires GIPR for hormone secretion from human islets. Nat Metab.

[54] Steuernagel L., Lam B.Y.H., Klemm P., Dowsett G.K.C., Bauder C.A., Tadross J.A. (2022). HypoMap—a unified single-cell gene expression atlas of the murine hypothalamus. Nat Metab.

[55] Adams J.M., Pei H., Sandoval D.A., Seeley R.J., Chang R.B., Liberles S.D. (2018). Liraglutide modulates appetite and body weight through glucagon-like peptide 1 receptor-expressing glutamatergic neurons. Diabetes.

[56] Fortin S.M., Lipsky R.K., Lhamo R., Chen J., Kim E., Borner T. (2020). GABA neurons in the nucleus tractus solitarius express GLP-1 receptors and mediate anorectic effects of liraglutide in rats. Sci Transl Med.

[57] Baggio L.L., Drucker D.J. (2007). Biology of incretins: GLP-1 and GIP. Gastroenterology.

[58] Mayendraraj A., Rosenkilde M.M., Gasbjerg L.S. (2022). GLP-1 and GIP receptor signaling in beta cells - a review of receptor interactions and co-stimulation. Peptides.

[59] Gabery S., Salinas C.G., Paulsen S.J., Ahnfelt-Rønne J., Alanentalo T., Baquero A.F. (2020). Semaglutide lowers body weight in rodents via distributed neural pathways. JCI Insight.

[60] Kaneko K., Fu Y., Lin H.Y., Cordonier E.L., Mo Q., Gao Y. (2019). Gut-derived GIP activates central Rap1 to impair neural leptin sensitivity during overnutrition. J Clin Invest.

[61] Mulvihill E.E., Drucker D.J. (2014). Pharmacology, physiology, and mechanisms of action of dipeptidyl peptidase-4 inhibitors. Endocr Rev.

[62] Al-Sabah S., Al-Fulaij M., Shaaban G., Ahmed H.A., Mann R.J., Donnelly D. (2014). The GIP receptor displays higher basal activity than the GLP-1 receptor but does not recruit GRK2 or Arrestin3 effectively. PLoS One.

[63] Gasbjerg L.S., Christensen M.B., Hartmann B., Lanng A.R., Sparre-Ulrich A.H., Gabe M.B.N. (2018). GIP(3-30)NH(2) is an efficacious GIP receptor antagonist in humans: a randomised, double-blinded, placebo-controlled, crossover study. Diabetologia.

[64] Jensen M.H., Sanni S.J., Riber D., Holst J.J., Rosenkilde M.M., Sparre-Ulrich A.H. (2024). AT-7687, a novel GIPR peptide antagonist, combined with a GLP-1 agonist, leads to enhanced weight loss and metabolic improvements in cynomolgus monkeys. Mol Metab.

