# Supplementary figures and images for: Specific loss of GIPR signaling in GABAergic neurons enhances GLP-1R agonist-induced body weight loss
Source: Mol Metab. 2024 Nov 26;95:102074. doi: 10.1016/j.molmet.2024.102074 (PMC11946504; doi:10.1016/j.molmet.2024.102074)

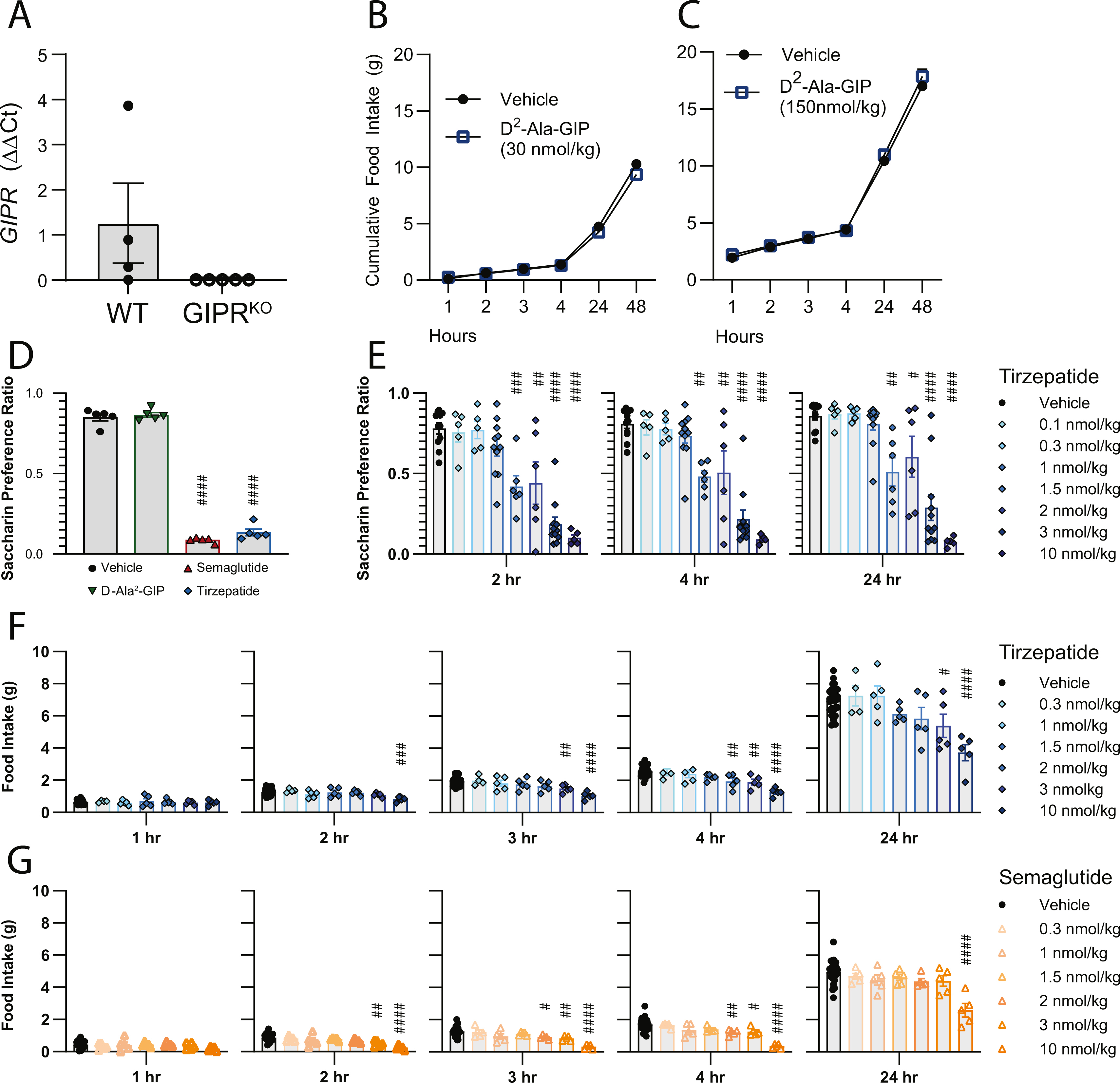

Supplement: Multimedia component 1 — D-Ala2-GIP characterization and dosage gradients. (A) qPCR of GIPR in white adipose tissue of GIPRKO mice (n = 4–5). Food intake measurements after a single dose of D-Ala2-GIP at (B) 30 nmol/kg or (C) 150 nmol/kg (B; n = 5–6, C; n = 5). (D) Conditioned taste aversion assay comparing D-Ala2-GIP to semaglutide and tirzepatide (n = 5). (E) Dosage gradient for tirzepatide in a conditioned taste aversion assay (n = 5–11). (F) Dosage gradient for tirzepatide in a short-term food intake assay (n = 4–30). (G) Dosage gradient for semaglutide in a short-term food intake assay (n = 5–30). Data in C–F was analyzed via 1-way ANOVA. Data are displayed as mean ± SEM. # indicates significance versus vehicle. #p < 0.05; ##p < 0.01; ###p < 0.001; ∗∗∗∗p < 0.0001. [file figs1.jpg]

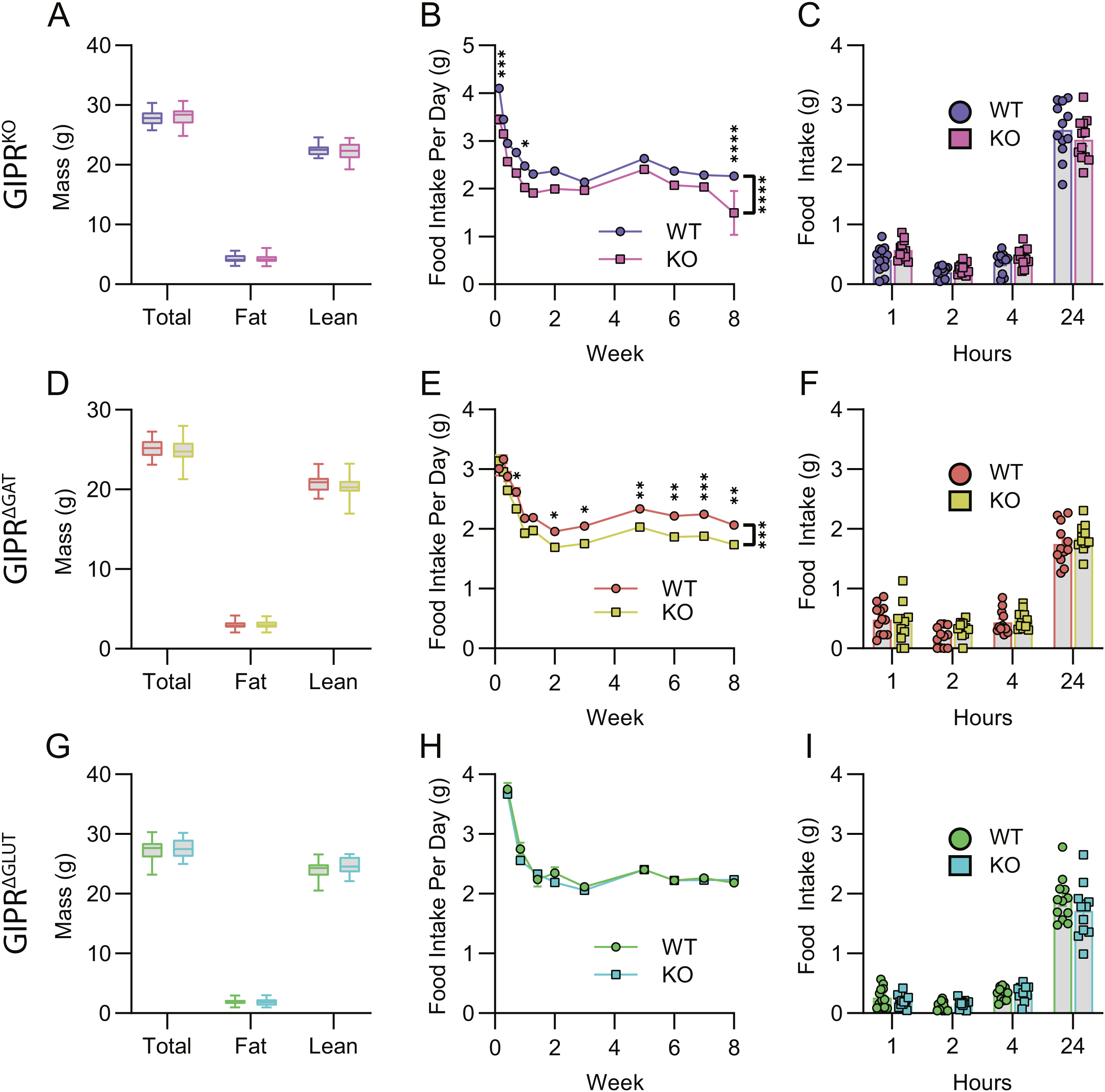

Supplement: Multimedia component 2 — Phenotypic characterization of GIPR KO models. (A) Body composition measurements of GIPRKO mice before HFD feeding. (B) Daily food intake for GIPRKO mice during HFD feeding (A–B; n = 30–31). (C) Fast/refeed assay for GIPRKO mice (n = 12). (D) Body composition measurements of GIPRΔGAT mice before HFD feeding. (E) Daily food intake for GIPRΔGAT mice during HFD feeding (D–E; n = 30). (F) Fast/refeed assay for GIPRΔGAT mice (n = 11–12). (G) Body composition measurements of GIPRΔGLUT mice before HFD feeding. (H) Daily food intake for GIPRΔGLUT mice during HFD feeding (G–H; n = 20–21). (I) Fast/refeed assay for GIPRΔGLUT mice (n = 12). Data in A, D, G were analyzed via two-tailed t-test. Data in B, C, E, F, H, and I were analyzed via a repeated measures 2-way ANOVA. ∗p < 0.05; ∗∗p < 0.01; ∗∗∗p < 0.001; ∗∗∗∗p < 0.0001. [file figs2.jpg]

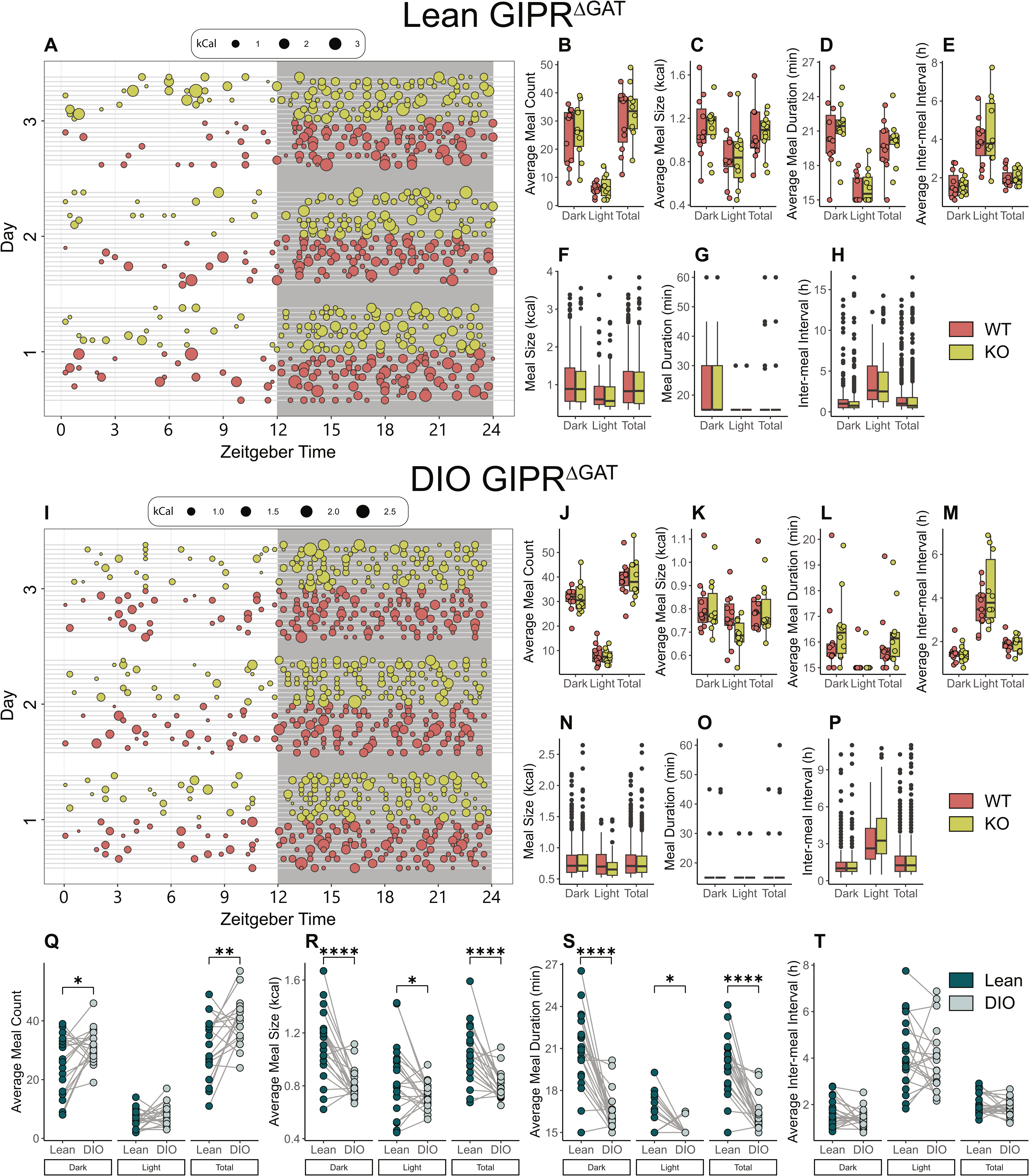

Supplement: Multimedia component 3 — Meal pattern analysis for GIPRΔGAT mice before and after DIO. (A) Meal patterns for three days in lean GIPRΔGAT mice. Each horizontal gray line represents a mouse. (B) Average meal count, (C) average meal size, (D) average meal duration and (E) average inter-meal interval per mouse (A–E; n = 10–11). (F) Pooled meal size, (G) meal duration, (H) and inter-meal interval. (I) Meal patterns for 3 days in DIO GIPRΔGAT mice. These are the same mice from A–H. (J) Average meal count, (K) average meal size, (L) average meal duration and (M) average inter-meal interval per mouse (I–M; n = 10–11). (N) Pooled meal size, (O) meal duration, (P) and inter-meal interval. (Q) Paired analyses of average meal count, (R) average meal size (S) average meal duration, (T) and average inter-meal interval of mice before and after induction of DIO via HFD feeding (n = 21). Data from B–H and J–P were analyzed via 2-way ANOVA. Data in Q, R, and T were analyzed via paired Student's t-test, and the data in S was analyzed via a Wilcoxon matched-pairs signed rank test. Data in B–H and J–P were displayed as a standard box and whisker plot: minimum, lower quartile, median, upper quartile, and maximum. ∗p < 0.05; ∗∗p < 0.01; ∗∗∗p < 0.001; ∗∗∗∗p < 0.0001. [file figs3.jpg]

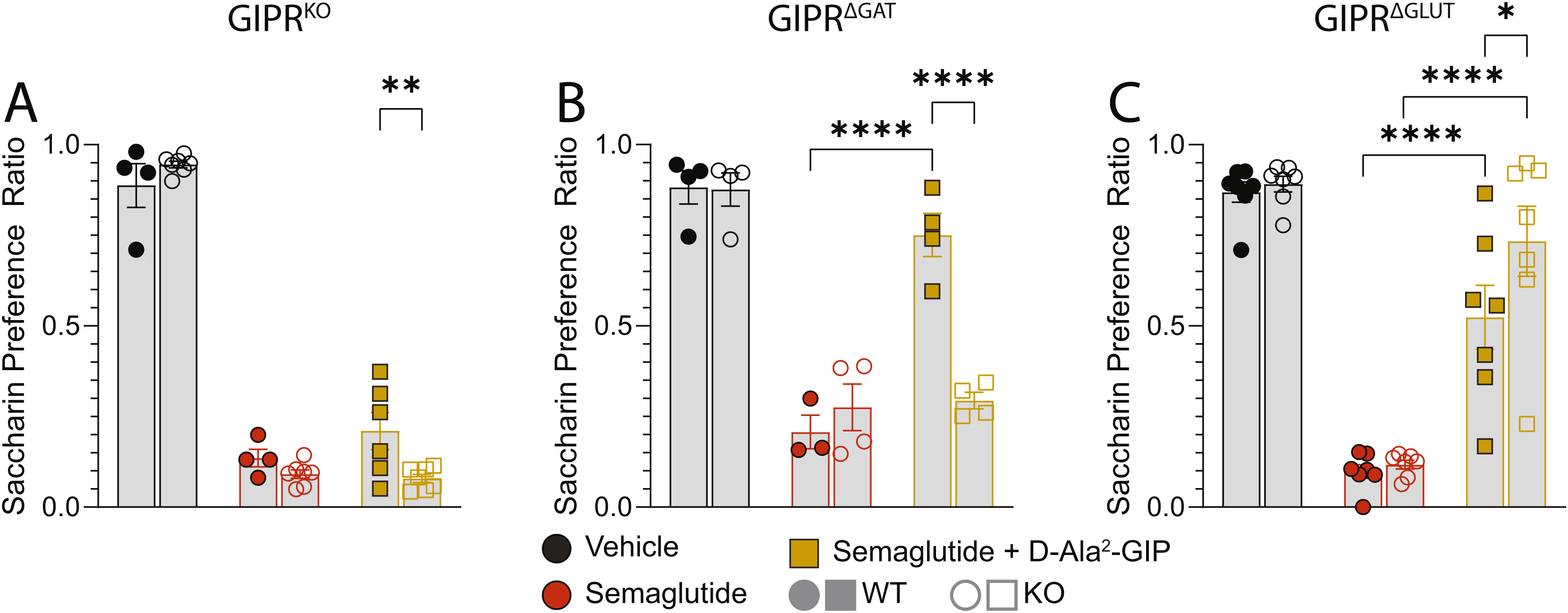

Supplement: Multimedia component 4 — Female conditioned taste aversion. (A) 24 h. measurement of saccharin preference ratio of female GIPRKO mice in a conditioned taste aversion assay (n = 4–7). (B) 24 h. measurement of saccharin preference ratio of female GIPRΔGAT mice in a conditioned taste aversion assay (n = 3–4). (C) 24 h. measurement of saccharin preference ratio of female GIPRΔGLUT mice in a conditioned taste aversion assay (n = 7). All data was analyzed via 2-way ANOVA. Data are displayed as mean ± SEM. ∗p < 0.05; ∗∗p < 0.01; ∗∗∗p < 0.001; ∗∗∗∗p < 0.0001. [file figs4.jpg]
